# iSCAN-V2: A One-Pot RT-RPA–CRISPR/Cas12b Assay for Point-of-Care SARS-CoV-2 Detection

**DOI:** 10.3389/fbioe.2021.800104

**Published:** 2022-01-21

**Authors:** Rashid Aman, Tin Marsic, Gundra Sivakrishna Rao, Ahmed Mahas, Zahir Ali, Madain Alsanea, Ahmed Al-Qahtani, Fatimah Alhamlan, Magdy Mahfouz

**Affiliations:** ^1^ Laboratory for Genome Engineering and Synthetic Biology, Division of Biological Sciences, 4700 King Abdullah University of Science and Technology, Thuwal, Saudi Arabia; ^2^ Department of Infection and Immunity, King Faisal Specialist Hospital and Research Centre, Riyadh, Saudi Arabia

**Keywords:** biosensing, CRISPR-Dx, POC Dx, COVID-19, SARS-CoV-2, RT-RPA, nucleic acid detection, CRISPR-Cas systems, Cas12b

## Abstract

Rapid, specific, and sensitive detection platforms are prerequisites for early pathogen detection to efficiently contain and control the spread of contagious diseases. Robust and portable point-of-care (POC) methods are indispensable for mass screening of SARS-CoV-2. Clustered regularly interspaced short palindromic repeats (CRISPR)/CRISPR-associated protein (Cas)-based nucleic acid detection technologies coupled with isothermal amplification methods provide a straightforward and easy-to-handle platform for detecting SARS-CoV-2 at POC, low-resource settings. Recently, we developed iSCAN, a two-pot system based on coupled loop-mediated isothermal amplification (LAMP) and CRISPR/Cas12a reactions. However, in two-pot systems, the tubes must be opened to conduct both reactions; two-pot systems thus have higher inherent risks of cross-contamination and a more cumbersome workflow. In this study, we developed and optimized iSCAN-V2, a one-pot reverse transcription-recombinase polymerase amplification (RT-RPA)-coupled CRISPR/Cas12b-based assay for SARS-CoV-2 detection, at a single temperature in less than an hour. Compared to Cas12a, Cas12b worked more efficiently in the iSCAN-V2 detection platform. We assessed and determined the critical factors, and present detailed guidelines and considerations for developing and establishing a one-pot assay. Clinical validation of our iSCAN-V2 detection module with reverse transcription-quantitative PCR (RT-qPCR) on patient samples showed 93.75% sensitivity and 100% specificity. Furthermore, we coupled our assay with a low-cost, commercially available fluorescence visualizer to enable its in-field deployment and use for SARS-CoV-2 detection. Taken together, our optimized iSCAN-V2 detection platform displays critical features of a POC molecular diagnostic device to enable mass-scale screening of SARS-CoV-2 in low-resource settings.

## Introduction

The outbreak of the novel coronavirus SARS-CoV-2, the causative agent of the COVID19 pandemic, poses a significant threat to all aspects of human life (Wu et al., 2020). Although preventive measures have been in place to minimize the number of new cases, mass screening is essential to identify and isolate SARS-CoV-2-infected individuals to limit virus spread and alleviate the burden on healthcare systems (Khan et al., 2020; Mercer and Salit, 2021). Additional implementation of effective measures, including testing, tracking, and tracing, is vital to limit transmission and control the spread of the virus (Güner et al., 2020).

Nucleic acid-based diagnostic systems are the most valued and effective methods for identifying a virus in a particular sample type. Although reverse transcription-quantitative PCR (RT-qPCR) remains the gold standard for detecting SARS-CoV-2 in patient samples, the need for well-equipped laboratories, expensive reagents, and trained personnel hinders its use in resource-limited areas. Therefore, there is a pressing need for portable and easy-to-deploy diagnostics at the point-of-care (POC) (Morshed et al., 2007; Sharma et al., 2015; Corman et al., 2020; Rezaei et al., 2020).

Recent advances in the field of diagnostics have highlighted the role of clustered regularly interspaced short palindromic repeats (CRISPR)/CRISPR-associated protein (Cas) technologies as a promising candidate for the development of user-friendly POC detection modules (Feng et al., 2020; Kaminski et al., 2021). The CRISPR/Cas target-specific endonuclease activities have been harnessed efficiently in genome engineering and virus interference technologies (Ran et al., 2013a; Ran et al., 2013b; Ali et al., 2015; Abudayyeh et al., 2017; Cox et al., 2017; Aman et al., 2018a; Ali et al., 2018; Aman et al., 2018b; Mahas and Mahfouz, 2018; Mahas et al., 2019; Ali et al., 2020a). The CRISPR/Cas type II system has been converted into several detection modalities such as FELUDA, CASLFA, and Vigilant (Wang et al., 2020; Azhar et al., 2021; Marsic et al., 2021). One of the most exciting features of several CRISPR/Cas types, such as types V and VI, is the activation of *in trans* promiscuous and collateral nucleic acid cleavage activity, following CRISPR RNA (crRNA)-based targeting and cleavage of a specific nucleic acid template. By harnessing the power of the collateral activity of these CRISPR/Cas enzymes, diverse nucleic acid detection modalities have been developed, including DETECTR, SHELOCK, iSCAN, SHINE, HOLMES, CDetection, APC-Cas, AIOD-CRISPR, and CRISPR-FDS (Gootenberg et al., 2017; Chen et al., 2018; Harrington et al., 2018; Kellner et al., 2019; Li et al., 2019; Teng et al., 2019; Arizti-Sanz et al., 2020; Ali et al., 2020b; Broughton et al., 2020; Ding et al., 2020; Joung et al., 2020; Cunningham et al., 2021; Ning et al., 2021). Systems that do not rely on nucleic acid pre-amplification have also been reported (Fozouni et al., 2021). Very recently, CRISPR/Cas type III systems have been harnessed for the development of pathogen detection platforms, thereby expanding the CRISPR/Cas diagnostic toolbox (Santiago-Frangos et al., 2021; Steens et al., 2021).

Using different reporters to harness the CRISPR enzymes collateral activity, subsequent to cis target cleavage activity, enables sensitive and specific signal readout for nucleic acids detection (Aman et al., 2020a; Kaminski et al., 2021). Fluorophore-labelled short nucleic acid reporters serve as a target for the *trans* collateral cleavage activity of the CRISPR/Cas endonuclease (Gootenberg et al., 2017; Li et al., 2018; Myhrvold et al., 2018). Depending on the type of fluorescent reporter used, the signal produced from CRISPR/Cas-dependent cleavage of the quenched fluorescent reporter is measured by a plate reader or a more straightforward fluorescent viewer device such as a p51 molecular viewer (Mahas et al., 2021a; Aman et al., 2020b). Another method of end-point result visualization utilizes FAM- or biotin-labelled reporters compatible with a commercially available lateral flow assay (LFA) (Kellner et al., 2019; Joung et al., 2020). A detailed overview of CRISPR/Cas-based diagnostics can be found in the review recently published by Kaminski et al. (2021).

Isothermal nucleic acid amplification methods like loop-mediated isothermal amplification (LAMP) and recombinase polymerase amplification (RPA) have been coupled with CRISPR/Cas-based systems to increase their sensitivity for nucleic acids diagnostics (Notomi et al., 2000; Piepenburg et al., 2006). To overcome the non-specific amplification and cross-contamination issues associated with isothermal amplification methods, there is a pressing need to develop one-pot amplification methods to bypass the repeated opening of reaction tubes after amplification to minimize workspace contamination. The coupling of CRISPR/Cas-based technologies with isothermal amplification provides an extra level of confirmation, enhances their specificity, and limits the non-specific amplification associated with these methods. To avoid cross-contamination and consequently the need for pre- and post-amplification working areas, another level of increased specificity can be secured by performing the amplification and CRISPR/Cas-based cleavage in a single tube (Joung et al., 2020). The single tube and single temperature requirement of such amplification platforms enable their use in POC settings. Moreover, because multiple reactions and components have to be conducted in the same tube with different enzyme requirements, optimizing the reaction chemistries and identifying the right enzymatic cocktail for the one-pot reaction, including a suitable reverse transcriptase, are prerequisites to enable their use at the POC (Ali et al., 2020b; Aman et al., 2020b; Mahas et al., 2021a).

**FIGURE 1:**
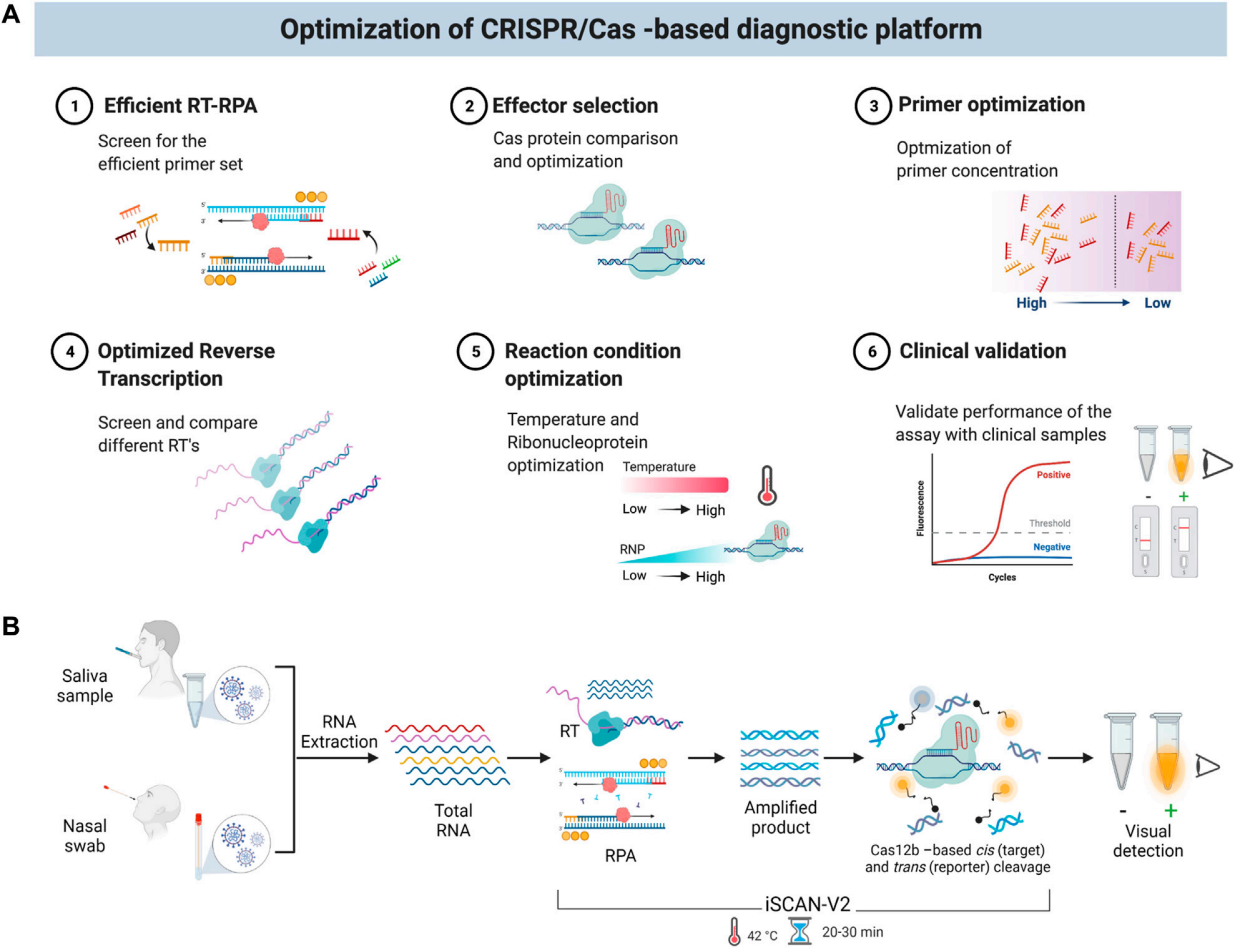
Schematic of the iSCAN-V2 one-pot assay. **(A)** Schematic of optimization steps for the iSCAN-V2 diagnostic platform. **(B)** An illustration of iSCAN-V2. Total RNA extracted from oropharyngeal or nasopharyngeal swabs was subjected to the iSCAN-V2 assay. The reverse transcription and amplification followed by Cas12b-crRNA-based targeted *cis* cleavage occurs in a single tube that activates Cas12b collateral activity. The collateral cleavage of the ssDNA-labelled HEX reporter provided in the same reaction produces a fluorescent signal that can be easily detected upon exposure to LEDs.

Here, we developed a SARS-CoV-2 detection system and optimized the reaction parameters to enable a sensitive and specific one-pot reaction. We systematically evaluated various reaction components and parameters (Figure 1A), including: 1) screening of different primer sets; 2) selection of the optimal Cas12 effector and ribonucleoprotein (RNP) complex concentration; 3) optimization of primer concentration; 4) optimization of the reverse transcription step; 5) evaluation of the optimal reaction temperature, and 6) clinical validation to show the utility in testing. Consequently, we report an efficient, isothermal, one-step/one-pot reverse transcription-recombinase polymerase amplification (RT-RPA) method coupled with the CRISPR/Cas12b system for the simple, specific, rapid, and sensitive detection of SARS-CoV-2 RNA in clinical samples. To enable its use in POC settings, we combined our detection module with a low-cost, commercially available P51 fluorescence viewer device to facilitate a quick signal readout suitable for in-field diagnostics. iSCAN-V2 works at a single isothermal temperature and can detect SARS-CoV-2 in patient samples in about 30 min, rendering a viable platform for large-scale screening in POC settings (Figure 1B). In conclusion, iSCAN-V2 exhibits the ASSURED (Accurate, Specific, Sensitive, User-friendly, Rapid, Equipment-free, and Deliverable to end-users) criteria defined by the World Health Organization for effective POC testing (Mabey et al., 2004).

## Results

### Establishment of iSCAN-V2

To facilitate and enable a POC diagnostic platform, we developed a single-pot assay where the amplification and detection steps occur in a single tube, in order to meet the ASSURED criteria, as described by the World Health Organization (Mabey et al., 2004; Land et al., 2019). We chose RPA as the isothermal amplification method due to its rapidity and low-temperature requirements. We tested several primer sets targeting a highly conserved region in the SARS-CoV-2 genome (the *N* gene) with crRNAs for Cas12a and Cas12b and tested their efficacy in a two-pot reaction by performing RT-RPA with synthetic SARS-CoV-2 RNA (Supplementary Figures S1A,B). In the case of Cas12a, we also designed a primer set amplifying a fragment of the *ORF1* gene. In a separate reaction, we confirmed the precision of the amplified product with the *cis* and *trans* cleavage properties of Cas12a and Cas12b. All primers and crRNAs showed robust performance, with both Cas enzymes resulting in high detection signals, confirming their specificity (Supplementary Figures S1A,B). Next, we tested all the primer sets in a one-pot system to select the most efficient set. Interestingly, among the different primer sets screened for Cas12a and Cas12b in the two-pot assay, only a single set of primers worked in the one-pot assay with both proteins (Supplementary Figures S1A,B). Moreover, our efficient primer set outperformed when compared to previously reported efficient RT-RPA primers (Qian et al., 2020). Therefore, we chose this working primer set for subsequent optimization steps.

**FIGURE 2:**
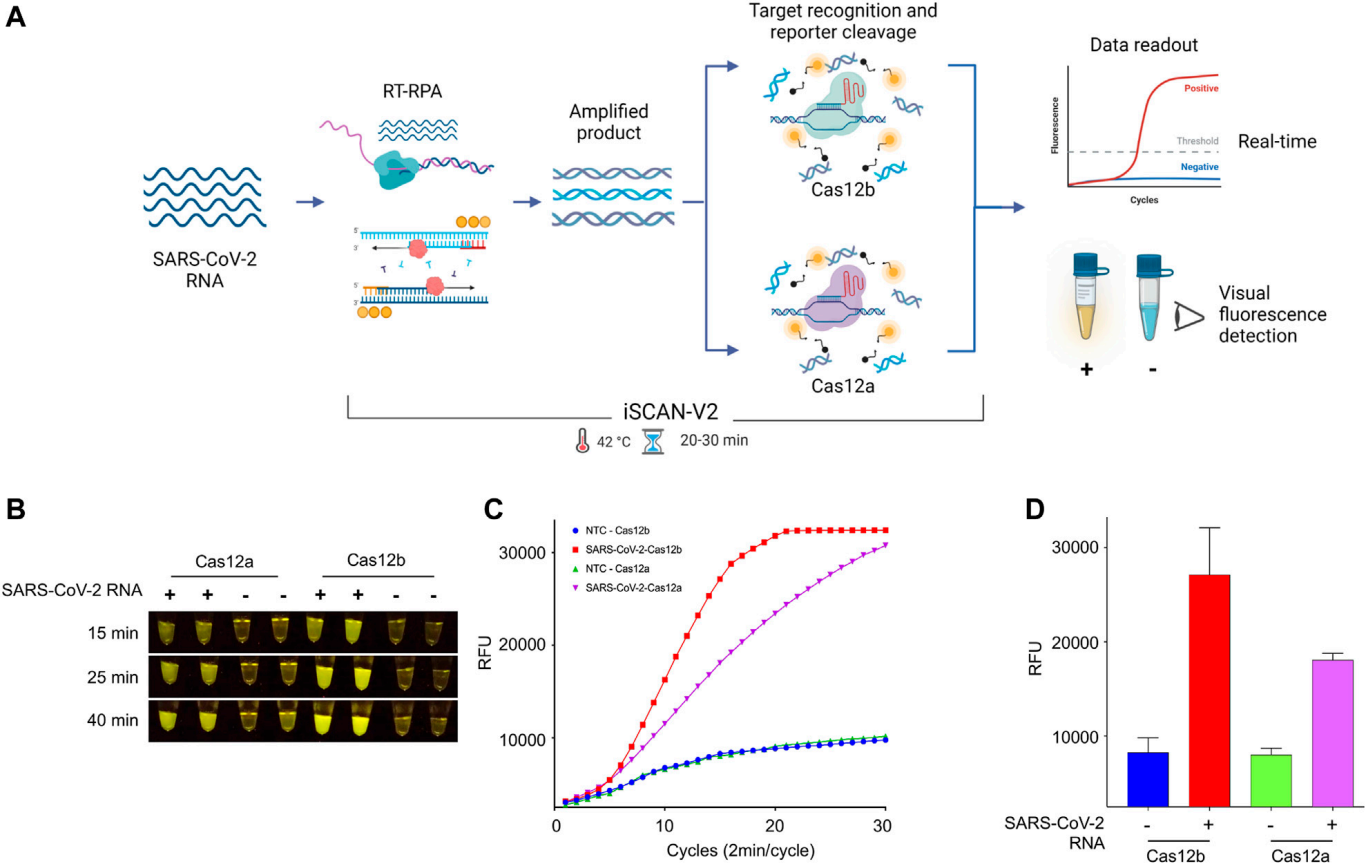
The iSCAN-V2 assay with Cas12b can efficiently detect SARS-CoV-2. **(A)** Schematic of CRISPR/Cas12a- and Cas12b-based detection of SARS-CoV-2. Total RNA was subjected to the iSCAN-V2 one-pot assay. The collateral cleavage of the ssDNA-labelled HEX reporter provided in the same reaction produces a fluorescent signal for visual detection. **(B)** iSCAN-V2-based detection of SARS-CoV-2. Synthetic SARS-CoV-2 RNA was subjected to the one-pot iSCAN-V2 platform using Cas12a or Cas12b. (+) indicates SARS-CoV-2 and (−) indicates the no-template control (nuclease-free water). For end-point fluorescence visualization of SARS-CoV-2 *N*-gene target sequence detection, samples were imaged in a P51 molecular fluorescence viewer. **(C)** Real-time detection of synthetic SARS-CoV-2 RNA. The iSCAN-V2 assay was performed to determine the real-time efficiency of Cas12a- and Cas12b-based target detection. Nuclease-free water was used as the no-template control (NTC). The intensity of the fluorescent signal was measured every 2 min for a period of 1 h using the HEX channel in a CFX96 Real-Time PCR System (Bio-Rad). Values are shown in the graph as means of 3 independent readings. **(D)** Graphical representation of iSCAN-V2 end-point detection performed with CRISPR/Cas12a and Cas12b. The iSCAN-V2 assay was performed as in **(B,C)**. (−) indicates the no-template control (nuclease-free water). The HEX channel was used to measure the fluorescent signal intensity with a CFX96 Real-Time PCR System (Bio-Rad). Values are shown in the graph as means ± SD (*n* = 3).

We next assessed the efficacy of Cas12a and Cas12b proteins in our iSCAN-V2 one-pot detection platform (Figure 2A). To this end, we performed the iSCAN-V2 assay with Cas12a and Cas12b proteins and compared their efficiencies by measuring end-point and real-time fluorescence signal intensities. We found Cas12b to be more efficient in the one-pot system than Cas12a when measuring the end-point fluorescence signal with a P51 visual fluorescence viewer (Figure 2B) after 15, 25, and 40 min. A clear visible difference was noticed after 15 and 25 min (Figure 2B).

Next, we evaluated the real-time performance of Cas12a and Cas12b in our iSCAN-V2 detection platform by measuring the fluorescence intensity every 2 min for a period of 1 h. The real-time data further indicated an early rise in the fluorescence signal for Cas12b as compared to Cas12a. Taken together with the end-point result, our data indicated that Cas12b performs more efficiently in the iSCAN-V2 detection platform when compared to Cas12a (Figures 2C,D). Therefore, we selected Cas12b for subsequent optimization steps.

### Evaluation of Assay Performance With Cas12b

**FIGURE 3:**
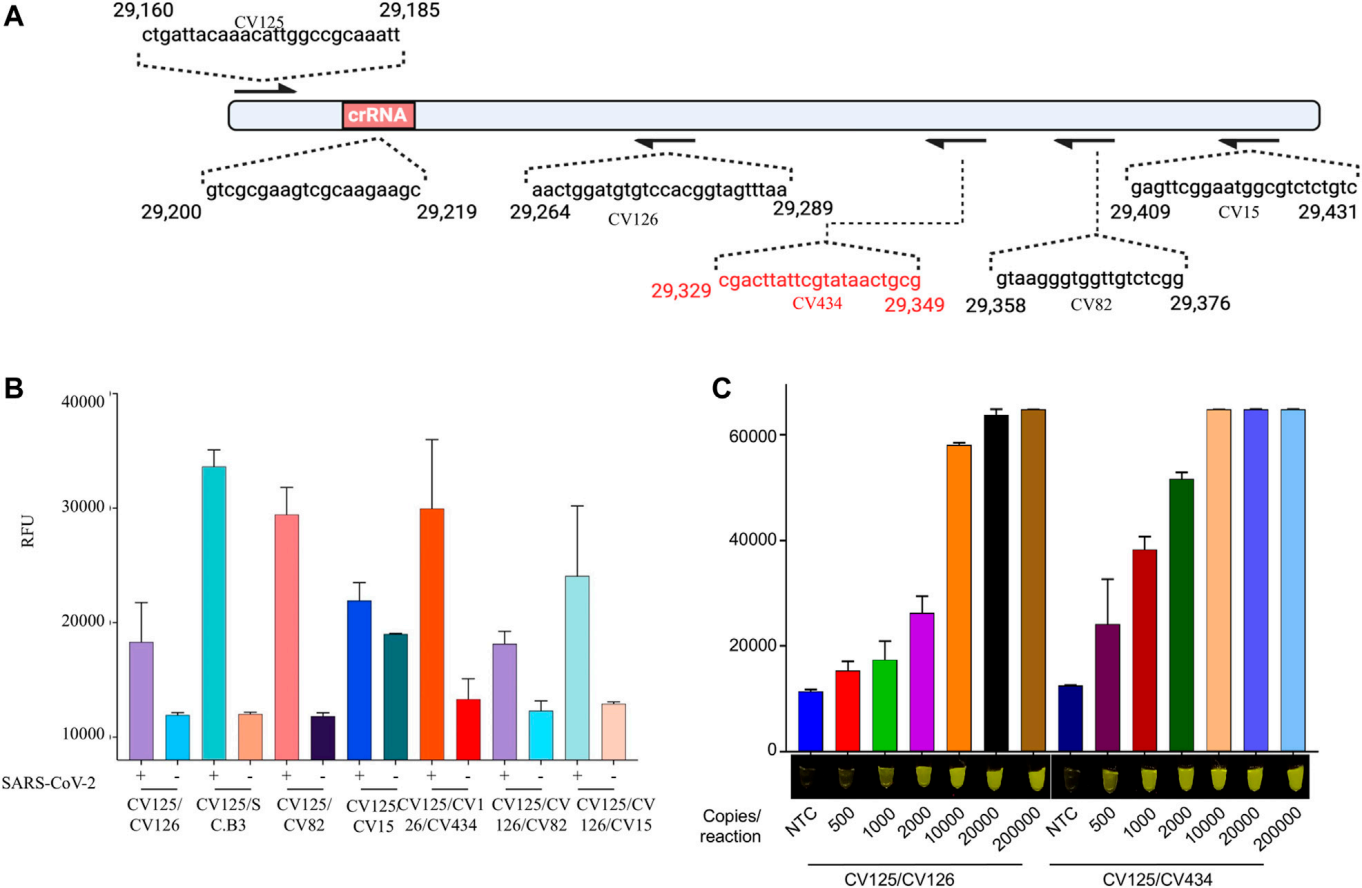
iSCAN-V2 demonstrates a high level of sensitivity for synthetic SARS-CoV-2 RNA detection. **(A)** Schematic of the SARS-CoV-2 N gene fragment. The forward (F) primer and multiple reverse (R) primers are represented by arrows and their sequence on the N gene fragment. **(B)** Limit of detection (LOD) determination of iSCAN-V2. Synthetic SARS-CoV-2 RNA (500, 1,000, 2000, 10,000, 20,000, and 200,000 copies/reaction) was subjected to the iSCAN-V2 detection platform using multiple primer sets. **(C)** LOD determination of iSCAN-V2. Synthetic SARS-CoV-2 RNA (50, 100, 200, 1,000, 2,000, and 20,000 copies/reaction) was subjected to the iSCAN-V2 detection platform using primer sets CV125/CV126 and CV125/CV434. CFX96 (Bio-Rad) end-point fluorescence (1 h) readouts were converted to graphical format using GraphPad Prism. For end-point fluorescence data presentation, error bars = means ± SD (*n* = 3). End-point fluorescence was imaged in a P51 molecular fluorescence viewer. For real-time representation, the intensity of the fluorescent signal was measured every 2 min for a period of 1 h. Values shown in the graphs are means of 3 independent readings. NTC, no template control.

To further evaluate the performance of the most promising primer set used with Cas12b, we performed an iSCAN-V2 assay with a range of SARS-CoV-2 copies per reaction and found the detection limit to be 40 copies/µl (Figure 3). To further boost the sensitivity, we screened several other reverse primers with the same forward primer (Figure 3A) and found two more sets of primers that performed superior to the primer set used in our previous optimization experiment (Figures 5B,D; Supplementary Figure S2). We also assumed that using multiple reverse primers with a single forward primer might enhance the reverse transcription and thus the limit of detection (LOD) of our assay. However, no significant increase in performance was observed when comparing the multiple reverse primers with the single reverse primer (Figures 3B,D; Supplementary Figures S3A,B). The increase in signal observed with primers CV125F and CV126R/CV434R compared to CV125F/CV126R was attributed mainly to the CV434R reverse primer. Moreover, we observed enhanced detection with this primer set when the activity was assessed with a range of synthetic SARS-CoV-2 RNA concentrations (Figure 3C; Supplementary Figure S2B). From these data, we concluded that CV125F and CV434R is the most efficient primer pair and selected this primer set for subsequent optimization steps.

### Efficient One-Pot Detection of SARS-CoV-2 With Superscript IV Reverse Transcriptase

The reverse transcription step is vital to enhance the sensitivity of the RT-RPA reaction. RNase H has an integral role in degrading the RNA strand in an RNA/DNA hybrid, thus accelerating the rate of reverse transcription (Supplementary Figure S3A) (Cerritelli et al., 2009). Therefore, to optimize the concentration of RNase H in our reaction setup, we performed the iSCAN-V2 assay using 0, 1, 2, and 4 units of RNase H per reaction (Supplementary Figures S3B,C). We found that using 1 unit of RNase H significantly affected the overall sensitivity of the iSCAN-V2 assay. We also observed impaired performance of the iSCAN-V2 assay when eliminating RNaseH from the reaction, which may be attributed to the highly stable DNA/RNA hybrid that inhibits the subsequent amplification step in the RT-RPA reaction.

**FIGURE 4:**
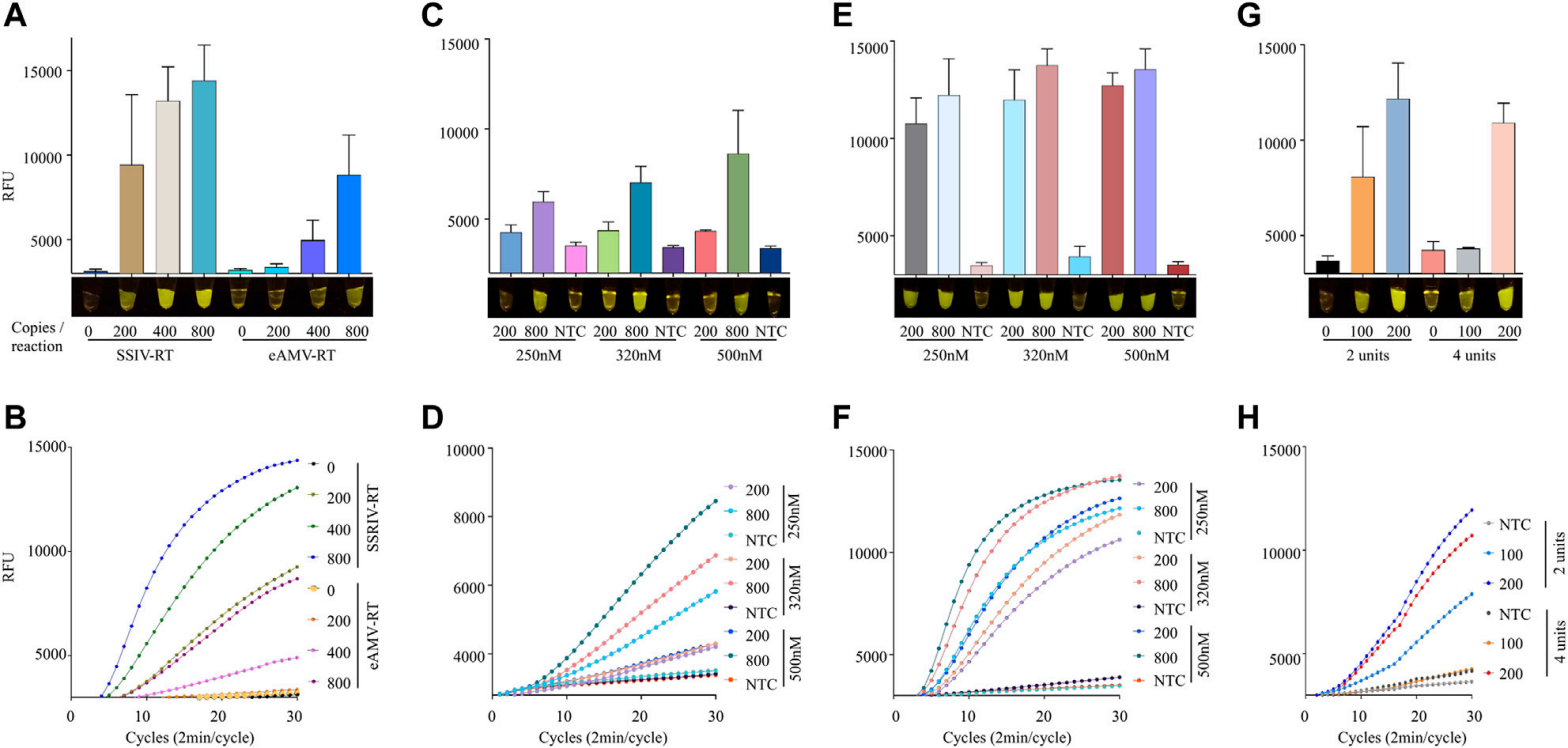
Efficient one-pot detection of synthetic SARS-CoV-2 RNA by iSCAN-V2 using SSIV-RT. **(A)** The efficacy of SSIV-RT and eAMV-RT was compared. Four units/reaction of eAMV-RT or SSIV-RT were used in iSCAN-V2 assays to detect synthetic SARS-CoV-2 RNA (200, 400, and 800 copies/reaction). The same primer concentration (320 nM) was used in all reactions. **(B)** Real-time representation of iSCAN-V2 assays using eAMV-RT and SSIV-RT shown in **(A)**. **(C)** The iSCAN-V2 assay was performed with eAMV-RT using different concentrations of primers (250, 320, and 500 nM) for the detection of synthetic SARS-CoV-2 RNA at 200 and 800 copies/reaction. **(D)** Real-time representation of iSCAN-V2 assays using eAMV-RT shown in **(C)**. **(E)** The iSCAN-V2 assay was performed with SSIV-RT using different concentrations of primers (250, 320, and 500 nM) for the detection of synthetic SARS-CoV-2 RNA at 200 and 800 copies/reaction. **(F)** Real-time representation of iSCAN-V2 assays using SSIV-RT shown in **(E)**. **(G)** The iSCAN-V2 assay was carried out with 2 or 4 units of SSIV-RT. H. Real-time representation of iSCAN-V2 assays using 2 or 4 units of SSIV-RT shown in **(G)**. CFX96 (Bio-Rad) end-point fluorescence readouts were converted to graphical format using GraphPad Prism. For end-point fluorescence data presentation, error bars = means ± SD (*n* = 3). End-point fluorescence was imaged in a P51 molecular fluorescence viewer. For real-time representation, the intensity of the fluorescent signal was measured every 2 min for a period of 1 h. Values shown in the graphs are means of 3 independent readings. NTC, no template control.

Next, we asked whether the choice of reverse transcriptase enzyme impacts the sensitivity of the iSCAN-V2 assay. Therefore, we tested Superscript IV reverse transcriptase (SSIV-RT) and enhanced avian reverse transcriptase (eAMV RT) with varying copy numbers of synthetic SARS-CoV-2 RNA to determine the precise LOD (Figures 4A,B). Our data showed a clear difference among these enzymes when comparing their end-point or real-time fluorescence output (Figures 4A,B). The end-point fluorescence data indicated that the iSCAN-V2 assay with SSIV-RT is able to detect as low as 8 copies/µl (200 copies/reaction) when compared to eAMV RT, which starts detecting SARS-CoV-2 RNA at 16 copies/µl (400 copies/reaction). To compare the efficiency of SSIV-RT and eAMV RT in real-time, we performed a one-pot assay with the HEX reporter and measured fluorescence every 2 min up to a total of 60 min. The real-time data confirmed the superior performance of SSIV-RT compared to eAMV RT. Our data indicated that our assay can detect 8 copies/µl with SSIV-RT and 32 copies/µl with eAMV RT (Figures 4A,B). We also performed an iSCAN-V2 assay with SSIII-RT enzyme as the reverse transcriptase, and compared its efficacy alongside SSIV-RT (Supplementary Figure S4A). Our data clearly indicated a more efficient detection of SARS-CoV-2 with SSIV-RT, specifically when comparing end-point fluorescence after 15 min of reaction time (Supplemental Figure S4A).

Next, we examined whether the detection sensitivity of these reverse transcriptases can be enhanced by modifying the primer concentration; therefore, we performed iSCAN-V2 assays with eAMV-RT and SSIV-RT using 250, 320, and 500 nM of primers with 200 or 800 copies of synthetic SARS-CoV-2 RNA per reaction. As anticipated, we observed a gradual increase in the fluorescence signal with higher primer concentrations when using 800 copies of synthetic SARS-CoV-2 RNA per reaction (Figures 4B,C). We concluded that increasing the primer concentration enhanced the overall sensitivity of our system.

The exact number of units of reverse transcriptase is crucial to obtain an efficient result (Robinson, 2015). Since SSIV-RT performed better compared to other reverse transcriptases, we tested three different concentrations of SSIV-RT using 100 and 200 copies/µl of synthetic SARS-CoV-2 RNA (Supplementary Figures S4B,C). Our data indicated that increasing the number of units of SSIV-RT at low copy number of SARS-CoV-2 inversely affected the overall detection. Therefore, we further reduced the number of units of SSIV-RT to ensure efficient detection of low copy numbers of SARS-CoV-2 RNA. We found that 2 units/reaction efficiently detected low copy numbers while performing comparably with higher copy numbers (Figures 3G,H).

### Optimization of Ribonucleoprotein Concentration and Temperature Conditions

**FIGURE 5:**
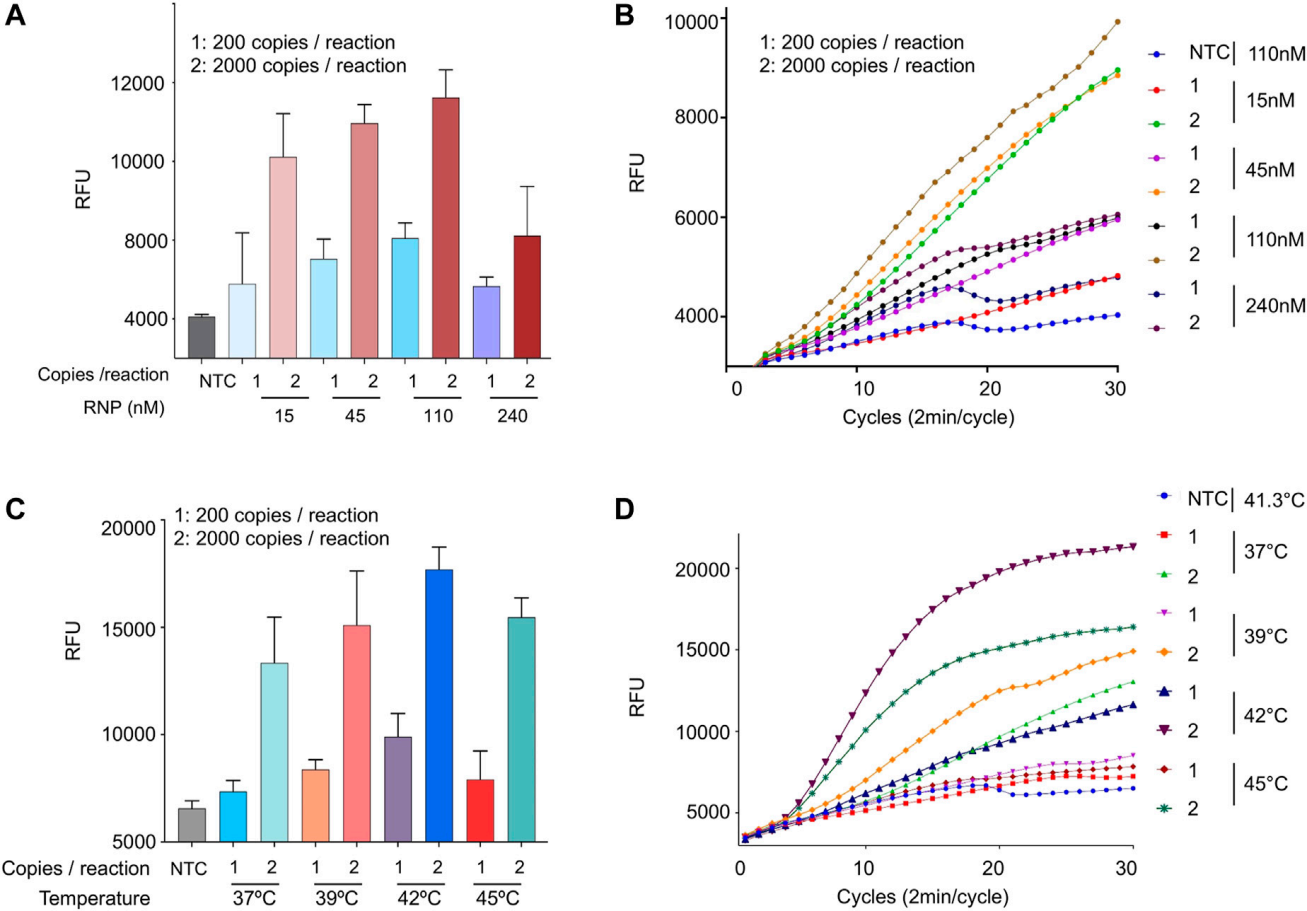
Optimization of RNP concentration and temperature conditions. **(A)** The effect of the RNP complex concentration on the iSCAN-V2 assay. The assay was performed with 15, 45, 110, and 240 nM of RNP. SARS-CoV-2 RNA at 800 copies/µl with 110 nM of RNP was used as a positive control. **(B)** Real-time representation of iSCAN-V2 assays performed with different concentrations of RNP as shown in **(A)**. **(C)** The effect of temperature on the iSCAN-V2 assay. The assay was performed using a temperature gradient (37, 39, 41.3, and 45°C). For the positive control, the assay was performed with 800 copies of SARS-CoV-2 RNA/µL at 41.3°C. **(D)** Real-time representation of iSCAN-V2 assays performed at different temperatures as shown in **(C)**. CFX96 (Bio-Rad) end-point fluorescence readouts were converted to graphical format using GraphPad Prism. For end-point fluorescence data presentation, error bars = means ± SD (*n* = 3). For real-time representation, the intensity of the fluorescent signal was measured every 2 min for a period of 1 hour. Values shown in the graphs are means of 3 independent readings. NTC, no template control.

Up until this point, we used 110 nM of Cas12b RNP in all our optimization assays. We wondered how varying the RNP concentration affects the sensitivity of the reaction. Therefore, we performed an iSCAN-V2 assay with RNP concentrations ranging from 15 to 240 nM per reaction (Figures 5A,B). We observed no significant improvement in performance at RNP concentrations higher than 110 nM. Therefore, we used the 110 nM concentration in all subsequent experiments.

RPA is known to work at a range of temperatures, optimally from 37 to 42°C (Lobato and O’Sullivan, 2018). Therefore, we performed a one-pot assay at a gradient of temperatures ranging from 37 to 45°C to screen for the best reaction conditions. Although RPA worked at all tested temperatures, a gradual increase in the signal intensity was observed until 42°C when using 200 or 2,000 copies of synthetic SARS-CoV-2 RNA per reaction. However, a sharp reduction in the signal intensity was observed at 45°C. The SSIV-RT and Cas12b enzymes are functional at high temperatures so the decrease in the overall sensitivity of iSCAN-V2 at 45°C is most likely attributed to the reduced performance of RPA components at high temperatures (Figures 5C,D).

### Limit of Detection and Clinical Validation of iSCAN-V2

**FIGURE 6:**
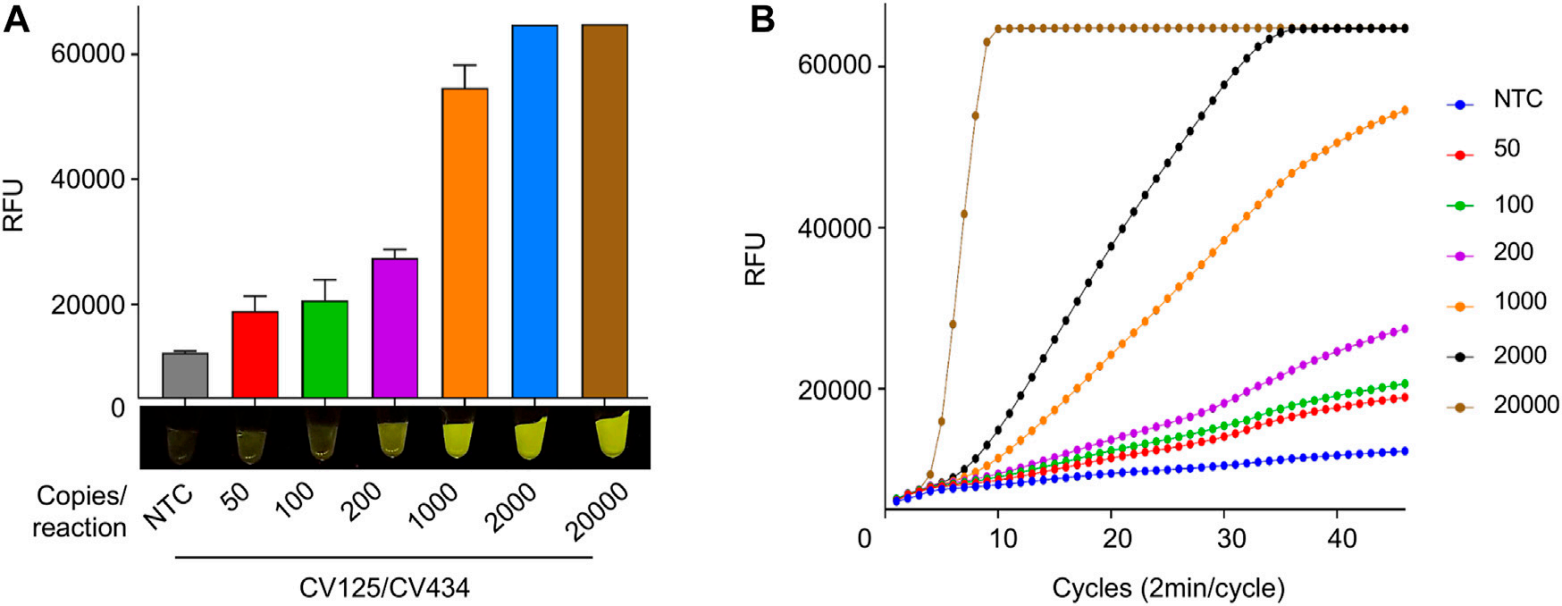
LOD of synthetic SARS-CoV-2 RNA using iSCAN-V2. **(A)** Synthetic SARS-CoV-2 RNA (50, 100, 200, 1,000, 2,000, and 20,000 copies/reaction) was subjected to the iSCAN-V2 detection platform using primer set CV125/CV434. **(B)** Real-time representation of iSCAN-V2 assays performed with different copy numbers of synthetic SARS-CoV-2 RNA as shown in A. CFX96 (Bio-Rad) end-point fluorescence (1 h) readouts were converted to graphical format using GraphPad Prism. For end-point fluorescence data presentation, error bars = means ± SD (*n* = 3). End-point fluorescence was imaged in a P51 molecular fluorescence viewer. For real-time representation, the intensity of the fluorescent signal was measured every 2 min for a period of 1 h. Values shown in the graph are means of 3 independent readings. NTC, no template control.

We next tested the analytical LOD of our optimized iSCAN-V2 assay using a serial dilution of synthetic SARS-CoV-2 RNA (Figures 6A,B). iSCAN-V2 was able to generate visible fluorescent signals down to 8 copies/µl of synthetic SARS-CoV-2 RNA, which is comparable to other reported isothermal amplification-coupled CRISPR/Cas-based detection platforms (Broughton et al., 2020).

**FIGURE 7:**
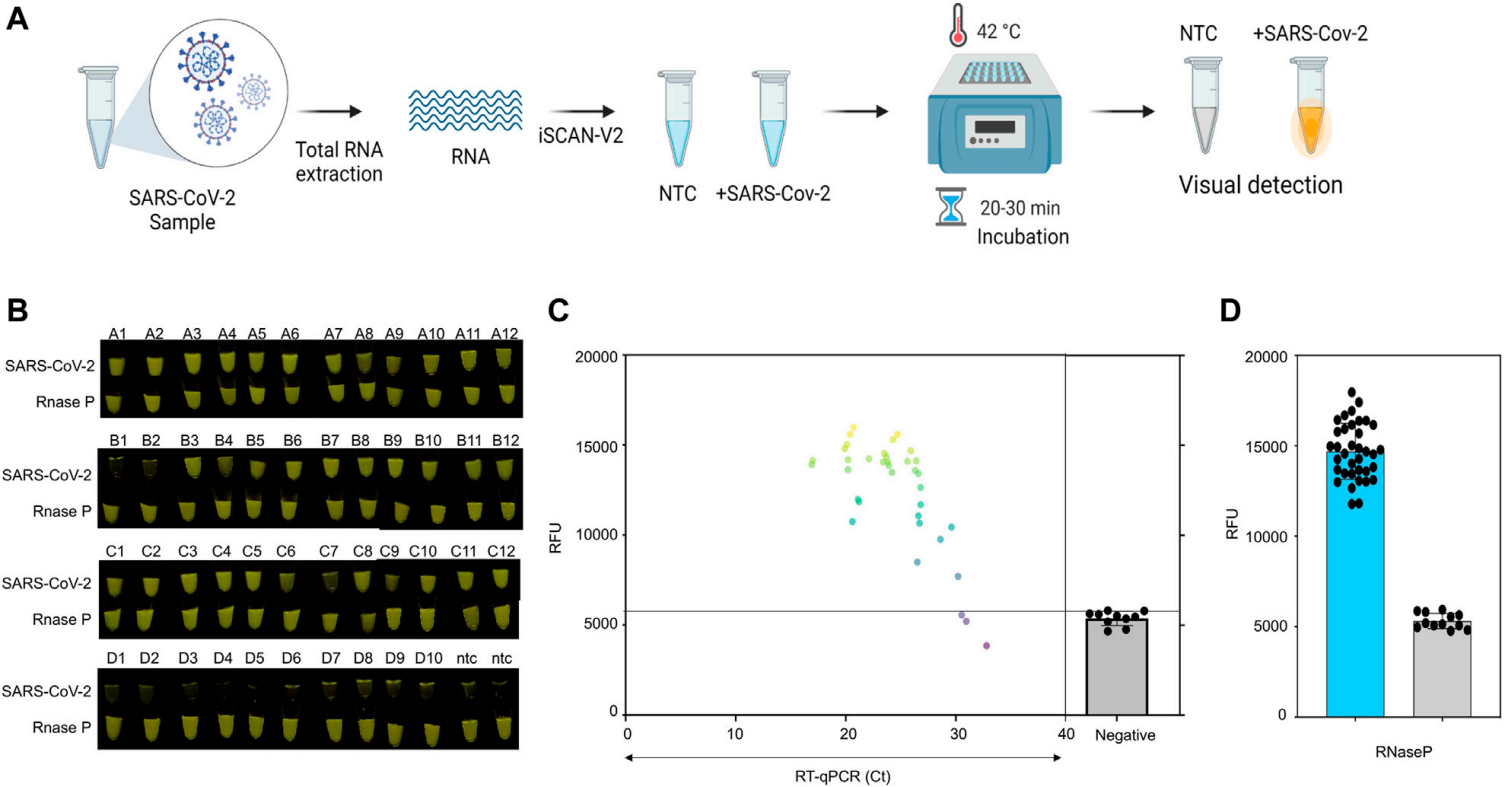
Clinical validation of iSCAN-V2. **(A)** Schematic of iSCAN-V2 validation for SARS-CoV-2 detection in clinical samples. **(B)** iSCAN-V2-based SARS-CoV-2 detection in clinical samples. Total RNA extracted from clinical samples [positive samples (*n* = 36) and negative samples (*n* = 10)] was subjected to the iSCAN-V2 platform for SARS-CoV-2 detection. *RNaseP* was used as the internal control for each sample. **(C)** Correlation of iSCAN-V2-based fluorescence readouts for SARS-CoV-2 detection with RT-qPCR cycle threshold (Ct) values in clinical samples. The grey line represents the background fluorescence in the negative samples. **(D)** End-point fluorescent readouts of *RNaseP* used as an internal control.

Subsequently, we validated the ability of iSCAN-V2 to detect SARS-CoV-2 in clinical samples containing total RNA extracted from nasopharyngeal swabs collected from suspected COVID-19 patients. Following total RNA extraction, positive and negative samples were identified by RT-qPCR according to the protocol approved by the Centers for Disease Control and Prevention. We performed the iSCAN-V2 assay on 36 positive and 12 negative clinical samples, including 2 no-template controls (NTC; nuclease-free water) as an experimental negative control (Figure 7; Supplementary Table S3). Out of 36 positive samples, iSCAN-V2 efficiently detected 33 positive samples, while all negative samples showed no signal, indicating the high specificity of our iSCAN-V2 detection platform (Figure 7B). Our iSCAN-V2 detection platform showed 93.75% concordance with the RT-qPCR positive and negative samples, which is comparable to previously reported isothermal-CRISPR/Cas-based diagnostic platforms. Overall, our data indicated that iSCAN-V2 could reliably and efficiently detect SARS-CoV-2 RNA in clinical samples with a Ct value of 30 or below in less than an hour (Figure 7C).

RNase P is used as an internal control to determine the integrity of clinical RNA samples (Ellis and Brown, 2009; Mautner et al., 2020; Ambrosi et al., 2021). We screened eight sets of RPA primers and six crRNAs to detect RNase P in total human RNA with the iSCAN-V2 assay. Out of 8 different sets screened, we found only 4 primer sets were compatible with our iSCAN-V2 detection module, with primer set 6 outperforming the others (Supplementary Figure S5). A saturation in the fluorescent signal was observed with primer set 6 in the first 30 min as indicated by end-point and real-time data (Supplementary Figures S5B,C). Therefore, we used this primer set to confirm the integrity of all the clinical samples used in this study (Figure 7D). All samples displayed a high fluorescent signal, confirming that all clinical samples used in this study contained sufficient levels of RNA (Figures 6B,D).

### iSCAN-V2 is Compatible With the Total RNA Quick Extraction Protocol

**FIGURE 8:**
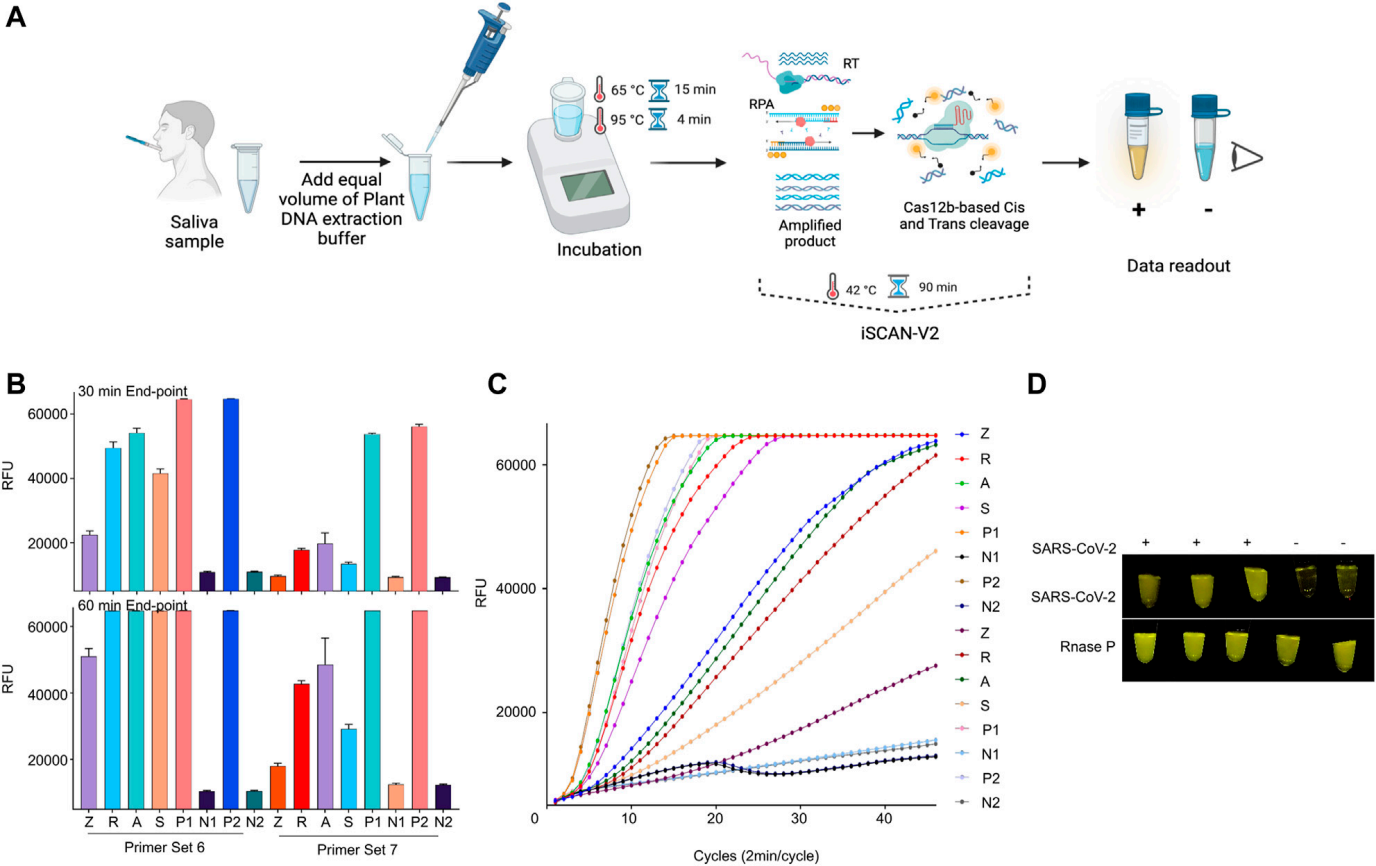
iSCAN-V2 is compatible with POC diagnostic setup. **(A)** Schematic of iSCAN-V2-based POC detection of SARS-CoV-2. **(B)** iSCAN-V2 one-pot assay for *RNaseP* detection. Total RNA was isolated from saliva samples using a quick extraction protocol. The extracted RNA samples (Z, R, A, and S) were subjected to the iSCAN-V2 platform for *RNaseP* detection. P1 and P2 indicate synthetic RNaseP RNA used as an experimental control. N1 and N2 indicate no template controls (NTC; nuclease-free water). **(C)** Real-time representation of iSCAN-V2 assays shown in **(B)**. The intensity of the fluorescent signal was measured every 2 min for a period of 30 min or 1 h. Values shown in the graphs are means of 3 independent readings. **(D)** iSCAN-V2-based detection of SARS-CoV-2 in SARS-CoV-2-spiked saliva samples. Total RNA extracted from the spiked saliva samples was subjected to the iSCAN-V2 platform for SARS-CoV-2 detection. *RNaseP* was used as an internal control for each sample. (+) indicates SARS-CoV-2-spiked saliva and (−) indicates non-spiked saliva. For end-point fluorescence visualization, samples were imaged in a P51 molecular fluorescence viewer.

One of the main limitations of a diagnostic assay to be employed at POC settings is the inability of quick sample processing. CRISPR/Cas-based diagnostic methods have been coupled with rapid total RNA extraction for human samples (Broughton et al., 2020; Ding et al., 2020; Smyrlaki et al., 2020). Therefore, we investigated whether our developed iSCAN-V2 platform is compatible with the previously reported quick RNA extraction buffer (Joung et al., 2020). Briefly, an equal volume of the extraction buffer is added to the saliva sample followed by heating at 95°C to release the total RNA and inactivate any enzymes (proteases) that may interfere with the RPA reaction (Figure 8A). To determine whether the quick RNA extraction method is feasible with our iSCAN-V2 one-pot assay, we collected saliva samples and subjected them to the quick extraction method. To substantiate this, we selected human *RNaseP* as a target and performed the iSCAN-V2 assay with the two most efficient primer (from primer screening, Supplementary Figures S5B,C) sets with their respective crRNAs (Figures 7B,C). The total RNA extracted from human cells with the commercial kit (Zymo Research) was used as a positive control in the iSCAN-V2 assay. The end-point and real-time data indicated that iSCAN-V2 could efficiently detect *RNaseP* in total RNA extracted via the quick extraction method. Moreover, while comparing the 30 min or 60 min end-point fluorescence data, we observed more efficient *RNaseP* detection with primer set 6 compared to primer set 7.

Due to the inaccessibility of real saliva samples from SARS-CoV-2-infected individuals, and to test the practicality of the quick RNA extraction method, we spiked saliva with synthetic SARS-CoV-2 RNA and subjected it to a quick RNA extraction procedure. Non-spiked saliva was processed and used as a negative control. The total RNA extracted was then subjected to the iSCAN-V2 assay. Our results demonstrated that iSCAN-V2 is compatible with the quick extraction method and can detect the SARS-CoV-2 genome when spiked into human saliva (Figure 7D).

Altogether, our data conclude that the iSCAN-V2 detection platform provides a simple, rapid, and easy-to-interpret method for SARS-CoV-2 detection and has the potential to be adopted as a POC diagnostic with minimal equipment and reagents. Using an inexpensive, hand-held fluorescence visualizer (P51 molecular fluorescence viewer) makes our iSCAN-V2 assay more amenable for use in POC settings by allowing simple visualization and interpretation of the end-point fluorescence data.

## Discussion

Based on our previously developed two-pot RT-LAMP-coupled CRISPR/Cas12a assay (iSCAN) for SARS-CoV-2 detection (Ali et al., 2020b), we converted the two-pot assay into a one-pot reaction to enable its practical use as a POC detection platform. One-pot isothermal amplification methods coupled with CRISPR/Cas systems are becoming the methods of choice in the CRISPR/Cas-based diagnostic field to avoid the risks of cross-contamination (Joung et al., 2020; Mahas et al., 2021b). Unlike two-pot assays that involve tube opening, and hence require pre- and post-amplification working areas to reduce cross-contamination, there is no tube-opening involved in one-pot assays as all reactions occur in the same tube.

During our optimization experiments, we found that primers played a crucial role in the overall performance of the system, which is mostly the case in any nucleic acid-based diagnostic platform. Primer efficiency is mostly attributed to efficient binding, and less or no secondary structure at the target area. Therefore, it is always recommended to design and screen multiple sets of primers. We found that only a few primer sets that worked efficiently in the two-pot assay also worked when coupled to Cas12 enzyme in the one-pot reaction. This highlights the need to screen multiple primer sets together with the Cas12 RNP to ensure that both of these reagents are tuned to simultaneously perform efficiently in one-pot reactions.

Since LAMP and RPA methods have stringent reaction condition requirements, selecting the suitable Cas12 effector compatible with isothermal amplification reaction chemistry is paramount. Selecting the appropriate Cas12 enzyme is crucial to the overall performance of the reaction. LbCas12a is a commonly used variant for genome engineering and diagnostics due to its high activity at 37°C. AapCas12b was identified as thermostable effector suitable for one-pot reactions at elevated temperatures (Broughton et al., 2020; Joung et al., 2020). However, AapCas12b also shows activity at 37°C. We suspected that properties intrinsic to each of these cas12 enzymes may have a critical role to play in one-pot reaction environment that has to be optimally fine tunned so that all reaction steps perform in a coordinated manner. We found that AapCas12b performed better than LbCas12a in the one-pot reaction, which might be attributed to specific properties of these two variants, such as temperature stability or more suitable enzyme kinetics.

We hypothesized that increasing the primer concentration might lead to improved sensitivity of the reaction. In order to test this, we conducted the assay using various primer concentrations. As expected, the real-time data from the iSCAN-V2 assay showed increased sensitivity when using a higher concentration of the primers (500 nM).

EAMV-RT is known for efficient cDNA synthesis from large mRNA templates with complex secondary structures (Mallet et al., 1995). Its other features and benefits include its greater sensitivity for low-abundance mRNA, transcription at high temperature, and efficient generation of full-length cDNA (up to 14 kb in size). SSIV-RT, on the other hand, is a customized product of the MMLV mutant with excellent robustness. In comparison to previous Superscript enzyme variants like SSIII-RT, SSIV-RT has improved inhibitor resistance, processivity, and reaction speed while retaining all the benefits of the previous enzymes, including high thermostability and efficient full-length cDNA synthesis with reduced RNaseH activity, which make it ideal for the rapid reverse transcription needed in diagnostic applications. SSIV-RT is designed to provide reliable, consistent, and fast cDNA synthesis in the presence of inhibitors found in a wide variety of samples that cause other currently available reverse transcriptases to perform inefficiently. As expected, SSIV-RT enabled efficient detection of 200 copies/reaction of synthetic SARS-CoV-2 RNA, demonstrating the utility of SSIV-RT to detect low copy numbers of SARS-CoV-2 RNA when used in the iSCAN-V2 assay when compared to other reverse transcriptases. Interestingly, we observed a decrease in performance when using a high number of units of SSIV-RT (above 4 units) at a low viral RNA copy number per reaction. In conclusion, our data indicate that SSIV-RT is the most suitable reverse transcriptase for our one-pot iSCAN-V2 assay specifically at low copies per reaction of SARS-CoV-2 RNA.

We also tested different RNP concentrations. We observed a gradual increase in the performance of the system up until 110 nM of RNP and a decrease in performance when using a higher concentration. The decline in the efficiency of the system could be attributed to the rapid degradation of the amplicons early on during the RT-RPA reaction in the presence of a high RNP concentration. We then performed the assay under different temperature conditions and found a gradual increase in performance with higher temperatures that peaked around 42°C, and a subsequent decrease at 45°C, which is likely associated with reduced activity of RPA reagents at elevated temperatures. These findings highlight the necessity of careful optimization of the components and their concentrations to establish a viable one-pot RT-RPA-coupled CRISPR/Cas-based detection module. When testing our fully optimized system on a synthetic target, we found the LOD to be 8 copies/µl, which is comparable to other diagnostic assays that are executed in multiple steps. Next, we validated the assay on clinical samples from COVID-19 patients and found a good agreement with RT-qPCR results at 93.75% sensitivity and 100% specificity. However, we noticed a decrease in agreement with samples with Ct values above 30.

Current limitations of the iSCAN-V2 system can be overcome by coupling it to a quick extraction protocol and RNA concentration step. As a proof of concept, we tested the compatibility of the iSCAN-V2 reaction with the quick RNA extraction protocol on mock samples. Our data suggest that iSCAN-V2 can efficiently detect synthetic SARS-CoV-2 RNA in spiked saliva samples. Furthermore, we envision coupling iSCAN-V2 to a mobile application that can aid in data interpretation and facilitate data sharing with centralized medical facilities.

In addition to developing a practical SARS-CoV-2 detection module, we highlighted the importance of optimizing different reaction parameters when developing a one-pot RT-RPA-coupled CRISPR/Cas-based detection assay. The resulting iSCAN-V2 module can be deployed in POC settings. Owing to its substantial time savings, robust specificity, and minimal equipment required, our iSCAN-V2 detection module can be easily adapted for large-scale virus screening in the field. We believe that our iSCAN-V2 platform exhibits the critical POC features to enable its use for mass-scale diagnostics for the current COVID-19 pandemic and future pandemics.

## Materials and Methods

### Protein Purification and Nucleic Acid Preparations

LbCas12a protein was purified as described by Chen et al. (2018), while AapCas12b protein was purified by Genscript. The synthetic SARS-CoV-2 viral RNA used in this manuscript was purchased from Twist Bioscience (cat. #102024). The original stock was first diluted to 10,000 copies per microliter, and further serially diluted into different concentrations based on the experimental requirements. Cas12a crRNAs were ordered as antisense strand ssDNA oligos appended with the T7-promoter in the forward primer. The ssDNA crRNA oligos were then annealed with T7-forward primer in 1× PCR buffer (−MgCl_2_; from Invitrogen) starting with denaturation at 95°C for 5 min followed by annealing at 5°C down to 4°C. The annealed product was used as a template for *in vitro* transcription. For Cas12b crRNAs, the long scaffold template was ordered as a sense strand ssDNA oligo appended with the T7 promoter at the 5′ end, and crRNAs were ordered as a reverse primer with a 21-bp complementary region. To incorporate the specific crRNA sequence and prepare the template for *in vitro* transcription, the scaffold was PCR amplified with a T7-forward and a crRNA-specific reverse primer. The clean PCR amplicons were purified (QIAquick PCR Purification Kit, QIAGEN) and *in vitro* transcribed using Transcript Aid T7 High Yield Transcription Kit (Thermo Scientific K0441) overnight at 37°C. According to manufacturer guidelines, the *in vitro* transcript products were then purified with Direct-zol RNA miniprep kit (R2050, Zymo Research). The purified RNA concentration was measured with a Nanodrop spectrophotometer (Thermo scientific) and diluted into working stocks of 5 µM.

### RPA Primers Design, Screening, and Reaction Conditions

Following the manufacturer’s instructions, RPA primers were designed with a range in length from 30 to 35 bp with melting temperatures of 55–67°C and ordered from IDT or Sigma. For RNaseP, eight RPA primer sets and five crRNAs spanning the whole sequence were designed and ordered from IDT. All RPA primers and crRNAs were screened for efficiency in the optimization experiments (Supplementary Tables S1, S2).

For all the RT-RPA reactions, the Twist-Dx kit from Twist Bioscience was used with slight modifications. Briefly, the RPA pellet was resuspended in 29.5 µl of buffer followed by addition of SSIV-RT (0.5 µl—or as stated otherwise), forward and reverse primers (1 µl each—500 nM final concentration), LbCas12a (0.5 µl–100 nM or as stated otherwise) or AapCas12b (0.3 µl–100 nM or as stated otherwise), crRNA (0.5 µM–110 nM or as stated otherwise), HEX reporter (3.75 µl–750 nM final concentration), and RNase H (0.5 µl). The reagents were properly mixed and divided equally at 21 µl into each tube. Then 2 µl of SARS-COV-2 RNA and MgOAC (magnesium acetate) was added to each tube and incubated at 42°C for 30 min to 1 h. For the clinical validation, a total of 5 µl of RNA extracted from SARS-CoV-2-infected patients was used and adjusted to a final volume of 25 µl with water. In the case of two–pot Cas12a or Cas12b assays, RT-RPA reactions were first performed without CRISPR/Cas proteins and HEX reporter. Subsequently, 2 µl of the RT-RPA reaction was added to the CRISPR/Cas-based detection assay. The HEX reporter signal was either measured with a Bio-Rad qPCR machine or manually observed with a P51 molecular fluorescence viewer (miniPCR. *P51™ Molecular Fluorescence Viewer*). For the specific information on the composition of iSCAN-V2 reaction, see Supplementary Table S4.

### Visual iSCAN-V2 Detection

For simple visualization, the collateral trans cleavage activity of Ca12a and Cas12b was measured by the HEX-labeled ssDNA reporter (/5HEX/TTTTTTT/3IABkFQ/) in the restriction reaction. The CRISPR/Cas-based collateral cleavage of ssDNA HEX reporters results in a signal easily visualized by light-emitting diodes. Complete iSCAN-V2 reaction tubes were transferred into the P51 Molecular Fluorescence Viewer (miniPCR), and the fluorescence signal was captured with a smartphone camera with an ISO setting of 200–400.

### Total RNA Extraction From Clinical Samples and Reverse Transcription Quantitative PCR of Clinical Samples

Oropharyngeal and nasopharyngeal swabs were collected and processed at the Department of Infection and Immunity at the King Faisal Specialist Hospital and Research Centre, Saudi Arabia. Following the manufacturer’s instruction, clinical samples were processed for total RNA extraction with RNA KingFisher Flex System and the MagMAX Viral/Pathogen Nucleic Acid Isolation Kit (cat. no. A42352). Total RNA was then converted into cDNA with SuperScript IV VILO master mix (Catalog # 11756500). The cDNA was then subjected to qPCR with an Applied Biosystems qPCR machine.

### Quick Total RNA Extraction From Saliva

An equal volume of saliva and quick DNA extraction buffer (QE09050, Lucigen) (50 µl each) was mixed and heated at 95°C to release total RNA and inactivate proteases that could negatively affect the RT-RPA-CRISPR-Cas12b (iSCAN-V2) reaction step. In the case of spiked saliva, a total of 20,000 copies of synthetic SARS-CoV-2 RNA was added to 50 µl of saliva and processed similarly. Out of the total 100, 5 µl of the total RNA sample was subjected to the iSCAN-V2 detection platform.

## Data Availability

The original contributions presented in the study are included in the article/Supplementary Material, further inquiries can be directed to the corresponding author.
