# Supplementary material for: iSCAN-V2: A One-Pot RT-RPA–CRISPR/Cas12b Assay for Point-of-Care SARS-CoV-2 Detection
Source: Front Bioeng Biotechnol. 2022 Jan 21;9:800104. doi: 10.3389/fbioe.2021.800104 (PMC8815761; doi:10.3389/fbioe.2021.800104)
Supplement: Supplementary file 1 [file DataSheet1.docx]

**Supplementary information**

**iSCAN-V2: a one-pot RT-RPA–CRISPR/Cas12b assay for point-of-care SARS-CoV-2 detection**

Rashid Aman^1^, Tin Marsic^1^, Gundra Sivakrishna Rao^1^, Ahmed Mahas^1^, Zahir Ali^1^, Madain Alsanea^2^, Ahmed Al-Qahtani^2^, Fatimah Alhamlan^2^, and Magdy Mahfouz^1,^ *

***Correspondence:** Magdy M. Mahfouz ([magdy.mahfouz@kaust.edu.sa](mailto:magdy.mahfouz@kaust.edu.sa))

Keywords: biosensing, CRISPR-Dx, POC Dx, biosensors, CRISPR, COVID-19, SARS-CoV-2, Superscript IV RT, RT-RPA, nucleic acid detection, CRISPR-Cas systems, Cas12b, molecular diagnostics


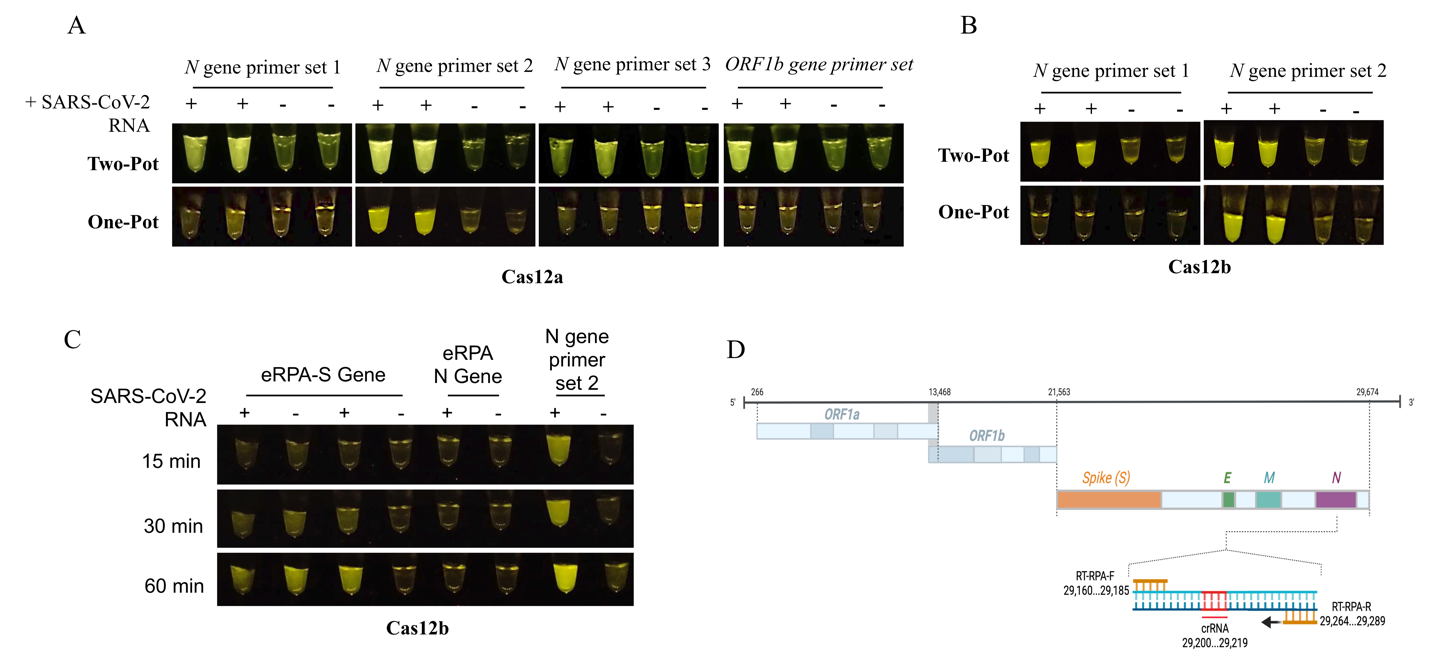


**Supplementary Figure 1: Primer efficiency screening for the iSCAN-V2** **assay**

1. Fluorescence visualization of the iSCAN-V2 detection assay performed with CRISPR/Cas12a. Three primer sets targeting the *N* gene and one targeting *ORF1b* with their respective crRNAs were screened for RT-RPA-based target amplification. (-) indicates the no-template control (nuclease-free water). The iSCAN-V2 assay was performed in two formats (two-pot and one-pot assays). For visual detection of the SARS-CoV-2 target sequence, samples were imaged in a P51 molecular fluorescence viewer.
2. iSCAN-V2 detection assay performed with CRISPR/Cas12b. *N* gene primer sets (primer set 1 and 2) for RT-RPA-based amplification and the respective crRNAs were used for target (*N* gene) detection in a one-pot iSCAN-V2 assay. (-) indicates the no-template control (nuclease-free water). The iSCAN-V2 assay was performed in two formats (two-pot and one-pot assays). For visual detection of the SARS-CoV-2 target sequence, samples were imaged in a P51 molecular fluorescence viewer.
3. iSCAN-V2 detection assay performed with CRISPR/Cas12b. *Spike* (*S*) gene and *N* gene primer sets for RT-RPA-based amplification and respective crRNAs were used for target detection in a one-pot iSCAN-V2 assay. (+) indicates the presence of synthetic SARS-CoV-2 RNA while (-) indicate its absence. For visual detection of the SARS-CoV-2 target sequence, samples were imaged in a P51 molecular fluorescence viewer.
4. The SARS-CoV-2 genome. The *N* gene sequence was selected for SARS-CoV-2 detection. RPA forward primer (RT-RPA-F) and reverse primer (RT-RPA-R) were designed to amplify a short fragment of the *N* gene. A crRNA labelled in red was designed for CRISPR/Cas12b-based targeted cleavage of the amplified product.


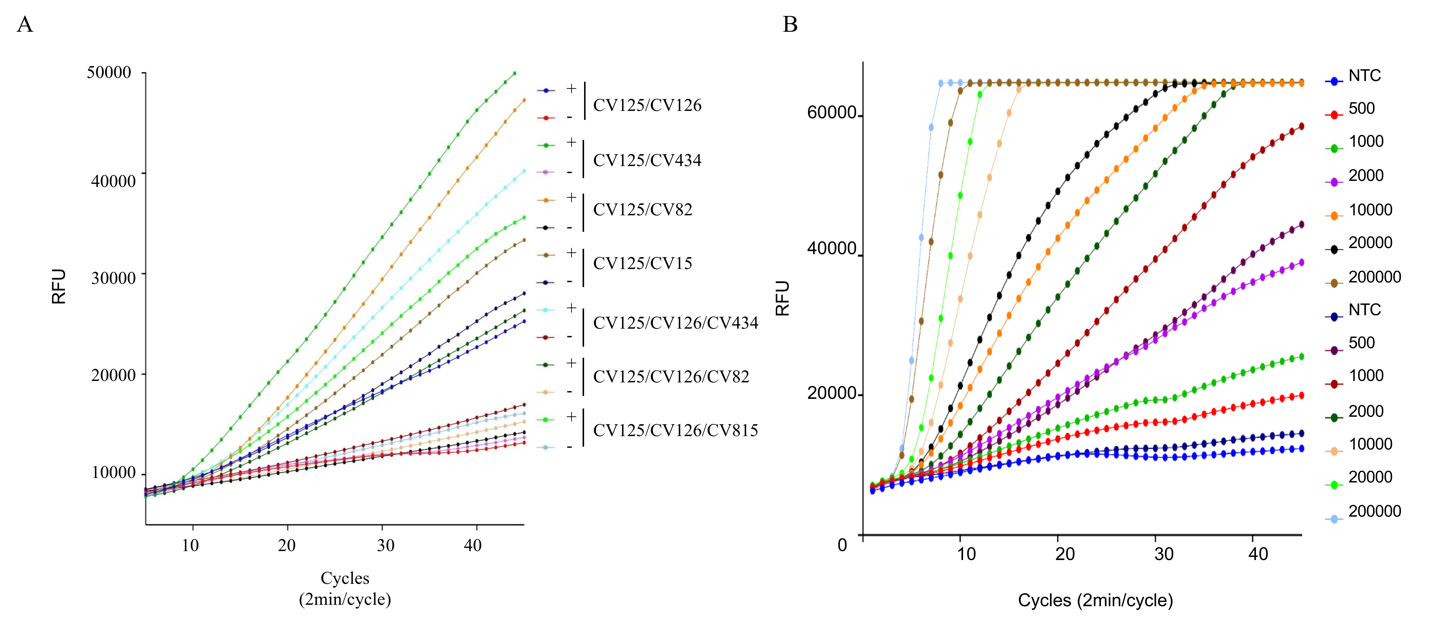


**Supplementary Figure 2: Screening for an efficient RNaseP primer set in the iSCAN-V2 assay**

1. iSCAN-V2 optimization via primer multiplexing. Synthetic SARS-CoV-2 RNA was subjected to the iSCAN-V2 detection platform using multiple primer sets.
2. Limit of detection (LOD) determination of iSCAN-V2. Synthetic SARS-CoV-2 RNA (500, 1000, 2000, 10,000, 20,000, and 200,000 copies/reaction) was subjected to the iSCAN-V2 detection platform using two primer sets, CV125/CV126 and CV125/CV434.

CFX96 (Bio-rad) end-point fluorescence readouts were converted to graphical format using GraphPad Prism. For end-point fluorescence data presentation, error bars = means ± SD (*n* = 3). For real-time representation, the intensity of the fluorescent signal was measured every 2 minutes for one hour. Values shown in the graphs are means of 3 independent readings.


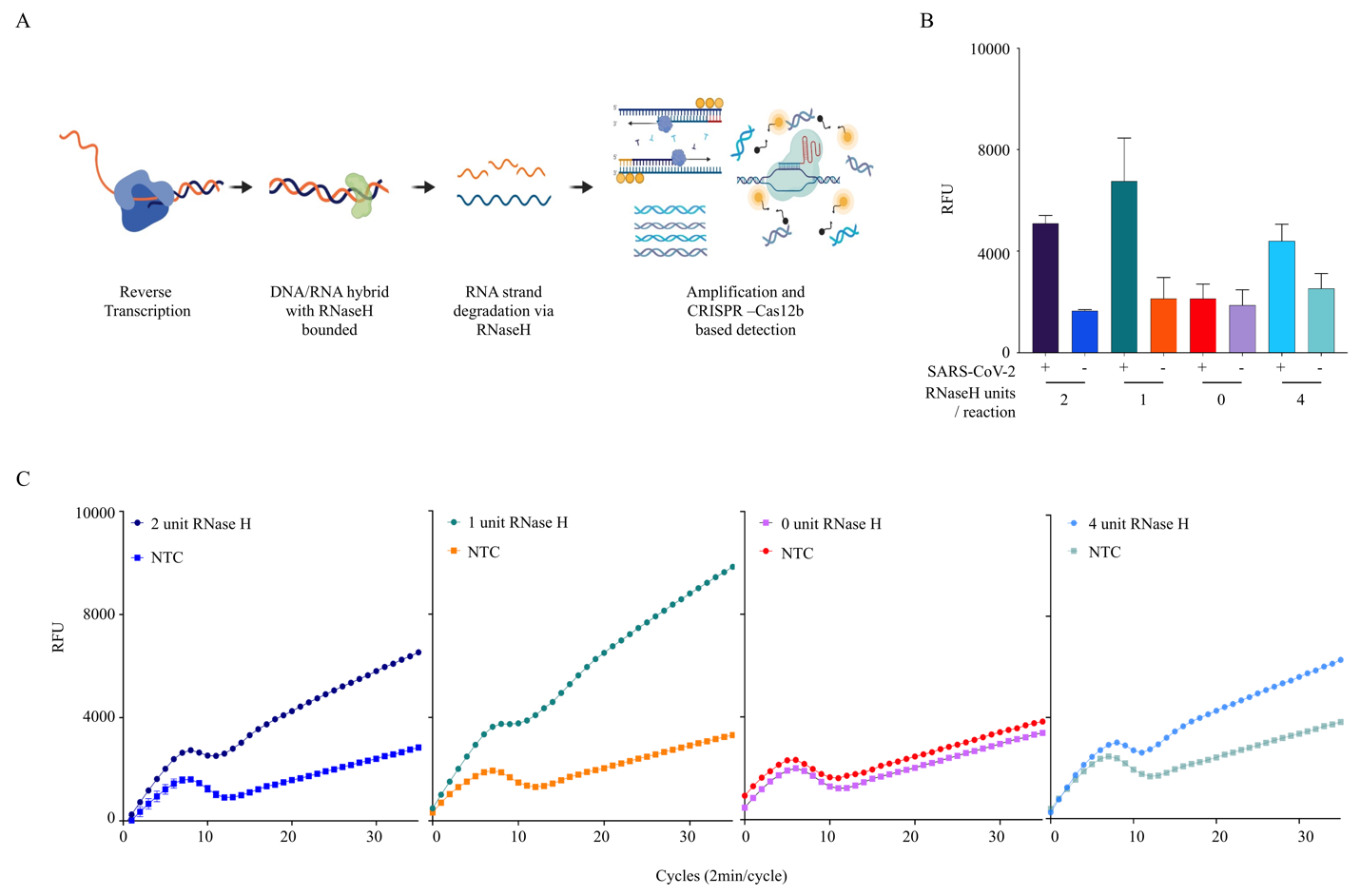


**Supplementary Figure 3: Optimization of the RNaseH concentration for the iSCAN-V2 assay**

1. Schematic of the RNaseH-coupled iSCAN-V2 assay. RNaseH was added to the one-pot iSCAN-V2 reaction to enhance RT-RPA.
2. RNaseH enhanced the RT-RPA reaction. Addition of 0.5 units/reaction of RNaseH enhanced the activity iSCAN-V2 for SARS-CoV-2 detection. RNaseH (0, 0.5, 1, and 2 units/reaction) was added to the iSCAN-V2 reaction. For results interpretation, CFX96 (Bio-Rad) end-point fluorescence readouts were converted into graphical format using GraphPad Prism. (+) indicates the presence of SARS-CoV-2 and (-) indicates the no-template control (nuclease-free water).
3. RNaseH improved synthetic SARS-CoV-2 RNA detection. Real-time representation of RNaseH (0, 0.5, 1, and 2 units/reaction)-based enhancement of SARS-CoV-2 detection. Real-time fluorescence of the reporter was measured every 2 minutes for a period of one hour with CFX96 (Bio-Rad). Values shown in the graphs are means of 3 independent readings.


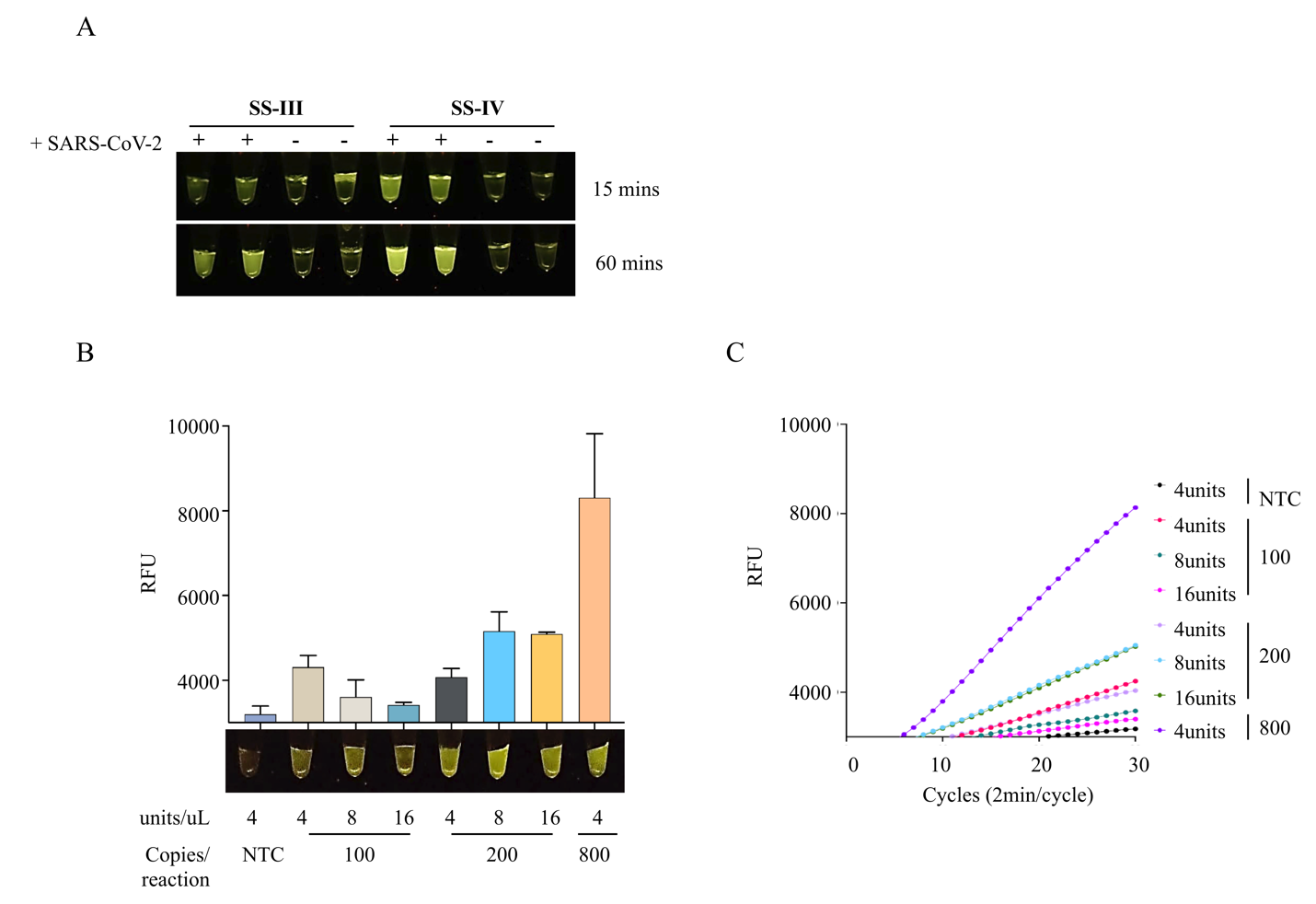


**Supplementary Figure 4: Superscript IV reverse transcriptase (SSIV-RT) performs better in iSCAN-V2**

1. Comparison of SSIII-RT and SSIV-RT efficiency in the iSCAN-V2 assay for SARS-CoV-2 detection. The iSCAN-V2 assay was performed with 4 units of SSIII-RT or SSIV-RT. End-point fluorescence (15 and 60 minutes) was imaged in a P51 molecular fluorescence viewer.
2. iSCAN-V2 assay optimization with SSIV-RT. The iSCAN-V2 assay was carried out with different (4, 8, and 16) units/reaction of SSIV-RT and different (100, 200, and 800) copies/reaction of synthetic SARS-COV-2 RNA. End-point fluorescence was imaged in a P51 molecular fluorescence viewer. CFX96 (Bio-Rad) end-point fluorescence readouts were converted for graphical representation using GraphPad Prism. Error bars = means ± SD (*n* = 3).
3. Real-time representation of iSCAN-V2-based synthetic SARS-CoV-2 RNA detection shown in B.


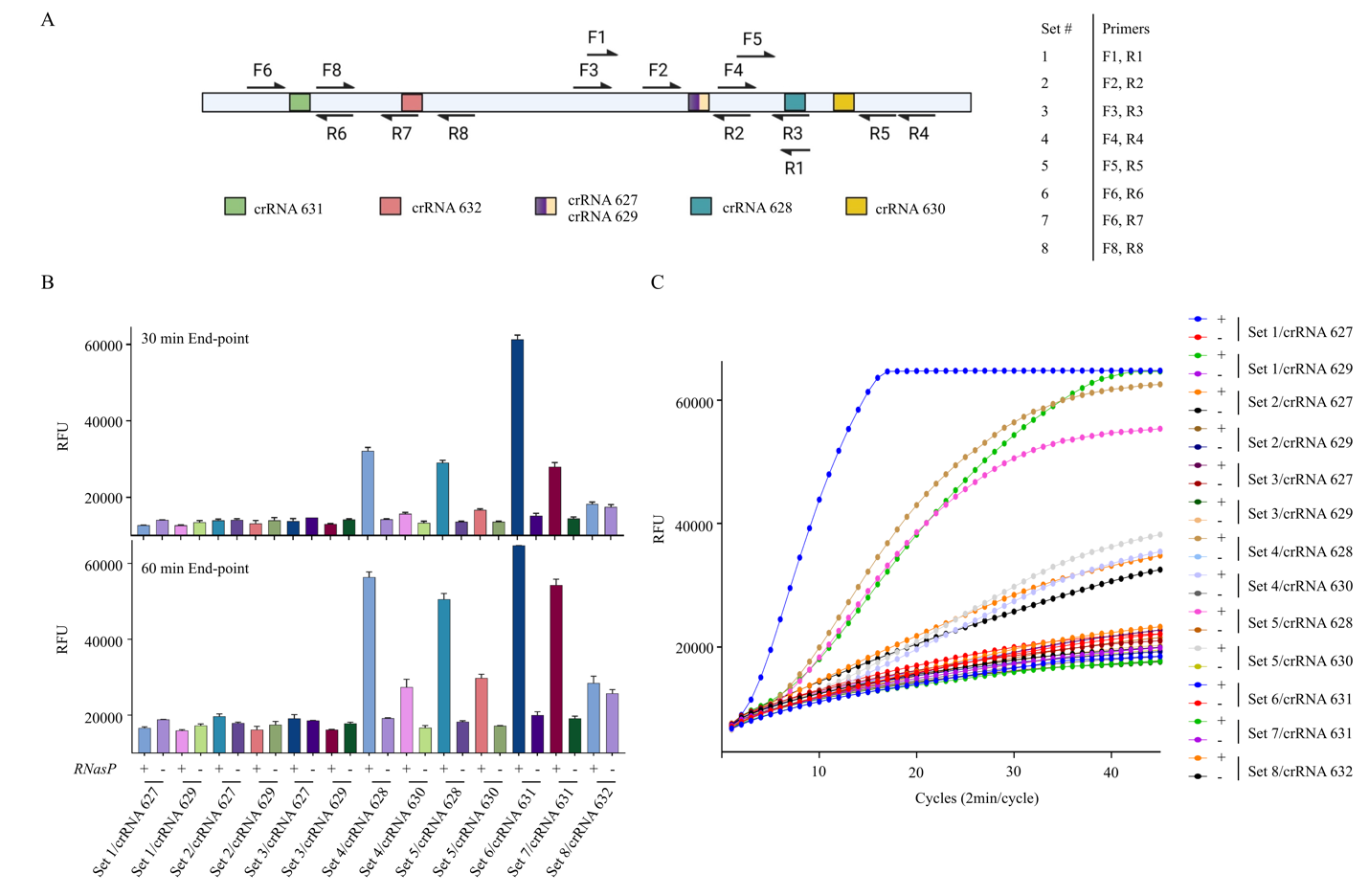


**Supplementary Figure 5: Screening for an efficient *RNaseP* primer set for the iSCAN-V2 assay**

1. Schematic of the *RNaseP* gene. The *RNaseP* gene was selected as an internal control for validating RNA integrity in clinical samples. Eight primer sets (indicated by arrows) along with five crRNAs (represented by colored boxes) were designed to detect synthetic *RNaseP* RNA.
2. Efficient *RNaseP* detection in the one-pot reaction. Synthetic *RNaseP* RNA was subjected to the iSCAN-V2 detection platform. Graphical representation of the iSCAN-V2 assay performed with different primers sets of *RNaseP* and their respective crRNAs. The graph represents end-point fluorescence data after 30 and 60 minutes of the iSCAN-V2 assay. (+) indicates the presence of *RNaseP* and (-) indicates its absence.
3. Real-time representation of iSCAN-V2-based detection of *RNaseP* using different primers and crRNAs sets.

CFX96 (Bio-Rad) end-point fluorescence readouts were converted to graphical format using GraphPad Prism. For end-point fluorescence data presentation, error bars = means ± SD (*n* = 3). For real-time representation, the intensity of the fluorescent signal was measured every 2 minutes for a period of one hour. Values shown in the graphs are means of 3 independent readings.

| Abbreviation | Name | Sequence | Purpose |
| --- | --- | --- | --- |
| *N* gene Primer set 1 | Forward primer | GGGGAACTTCTCCTGCTAGAAT | Used with Cas12a and Cas12b |
|  | Reverse primer | CAGACATTTTGCTCTCAAGCTG |  |
| *N* gene Primer set 2  (CV125/CV126) | Forward primer | ctgattacaaacattggccgcaaatt | Used with Cas12a and Cas12b |
|  | Reverse primer | aatttgatggcacctgtgtaggtcaa |  |
| *N* gene Primer set 3 | Forward primer | agctaccagacgaattcgtggtggtgacgg | Used with Cas12a |
|  | Reverse primer | acgattgcagcattgttagcaggattgcgg |  |
| *Orf1b* primer set | Forward primer | CCCTGTGGGTTTTACACTTAA | Used with Cas12a |
|  | Reverse primer | ACGA TTGTGCA TCAGCTGA |  |
| CV434 | Reverse primer | GCGTCAATATGCTTATTCAGC | Used as reverse primer with *N* gene forward primer (Set 2 , CV125) to amplify the N gene fragment |
| CV82 | Reverse primer | ggctctgttggtgggaatg |  |
| CV15 | Reverse primer | ctgtctctgcggtaaggcttgag |  |
| CV609 | RNase P gene-F1 | tggagccagagaccgacaca | RnaseP primers used in screening for efficient pair |
| CV610 | RNase P gene-R1 | acatggctctggtccgaggt |  |
| CV611 | RT-RPA-RnaseP-F2 | ggatccgcaacaactcagccatccacatcc |  |
| CV612 | RT-RPA-RnaseP-F3 | cttgttgatgagctggagccagagaccgacac |  |
| CV613 | RT-RPA-RnaseP-F4 | ctccacttatcccctccgtgatatggctcttcg |  |
| CV614 | RT-RPA-RnaseP-F5 | gtgatatggctcttcgcatgctgagtactgg |  |
| CV615 | RT-RPA-RnaseP-F6 | gctggctgcttgcacccacatccttctttc |  |
| CV616 | RT-RPA-RnaseP-F8 | gttacttgggctggccggcgaacccttg |  |
| CV617 | RT-RPA-RnaseP-R2 | catatcacggaggggataagtggaggagtg |  |
| CV618 | RT-RPA-RnaseP-R3 | cttacatggctctggtccgaggtccagtac |  |
| CV619 | RT-RPA-RnaseP-R4 | cgcatacacacactcaggaaggcccactaactg |  |
| CV620 | RT-RPA-RnaseP-R5 | gcccaccaagagacaattacccccaccctc |  |
| CV621 | RT-RPA-RnaseP-R6 | caagggttcgccggccagcccaagtaa |  |
| CV622 | RT-RPA-RnaseP-R7 | ccatgctgtgtgcctctcttcgttccacag |  |
| CV623 | RT-RPA-RnaseP-R8 | cctaagggtgtattccactggatccagttcagcc |  |

**Table S1: Primers used in this study.**

**Table S2: crRNAs and fluorescent reporter used in this study.**

| Abbreviation | Name | Sequence | Purpose |
| --- | --- | --- | --- |
| CV254 | T7-3G IVT primer-1 | GAAATTAATACGACTCACTATAGGG | T7 forward primer |
| CV431 | N -N2 Zhang gene Cas12a gRNA | GAACGCTGAAGCGCTGGGGGATCTACACTTAGTAGAAATTACCCTATAGTGAGTCGTATTAATTTC | Cas12a-*N* gene crRNA for CV125/cv126 region |
| CV456 | N -N2 Zhang gene Cas12b gRNA | CAGCGCTTCAGCGTTCTTCGGTGCCACTTCTCAGATTTGAG | Cas12b-*N* gene crRNA for CV125/cv126 region |
| CV241 | AaCas12b sgRNA scafold-Top | GAAATTAATACGACTCACTATAGGGTCTAGAGGACAGAATTTTTCAACGGGTGTGCCAATGGCCACTTTCCAGGTGGCAAAGCCCGTTGAGCTTCtcaaatctgaGAAGTGGCAC | Cas12b scaffold |
| CV247 | N gene-3/A Cas12 gRNA-R | ggacttccctatggtgctaaATCTACACTTAGTAGAAATTACCCTATAGTGAGTCGTATTAATTTC | Cas12a N gene crRNA for RPA N3-1 primers |
| CV627 | Cas12b-RnaseP-gRNA1 | agggtcacacccaagtaattGTGCCACTTCtcagatttgaG | RNaseP crRNAs used in the iSCAN-V2 assay to screen for efficiency |
| CV628 | Cas12b-RnaseP-gRNA2 | ctcggaccagagccatgtaaGTGCCACTTCtcagatttgaG |  |
| CV629 | Cas12b-RnaseP-gRNA3 | aattacttgggtgtgaccctGTGCCACTTCtcagatttgaG |  |
| CV630 | Cas12b-RnaseP-gRNA4 | gagtcctttgggcttccaggGTGCCACTTCtcagatttgaG |  |
| CV631 | Cas12b-RnaseP-gRNA5 | ccgcgaccccaggtcccagaGTGCCACTTCtcagatttgaG |  |
| CV632 | Cas12b-RnaseP-gRNA6 | gagaggcacacagcatggcaGTGCCACTTCtcagatttgaG |  |
| CV457 | ESSENTIAL Cas12HEX reporter-1 | /5HEX/TT TTT TT/3IABkFQ/ | HEX-labeled ssDNA reporter sequence |

**Table S3: Ct values of the clinical samples used in this study**

| Sample Number | CT values for SARS-CoV-2 | Random fluorescence signal as measured by CFX96 (Bio-Rad) |
| --- | --- | --- |
| 1 | 20.2 | 13630.95 |
| 2 | 23.8 | 14050.22 |
| 3 | 20.4 | 15594.43 |
| 4 | 23.9 | 13856.65 |
| 5 | 21.2 | 11841.42 |
| 6 | 20.2 | 14184.92 |
| 7 | 20.6 | 10741.53 |
| 8 | 26.5 | 8488.768 |
| 9 | 24.2 | 13470.45 |
| 10 | 26.3 | 13583.96 |
| 11 | 22.1 | 14230.25 |
| 12 | 26.7 | 10652.21 |
| 13 | 32.79 | 3847.848 |
| 14 | 30.95 | 5209.065 |
| 15 | 19.9 | 14791.96 |
| 16 | 26.8 | 11682.01 |
| 17 | 23.4 | 14055.41 |
| 18 | 23.5 | 14531.11 |
| 19 | 25.9 | 14682.74 |
| 20 | 26.8 | 12641.87 |
| 21 | 26.6 | 13409.62 |
| 22 | 29.6 | 10431.91 |
| 23 | 28.6 | 9752.904 |
| 24 | 21.1 | 11978.74 |
| 25 | 23.7 | 14334.44 |
| 26 | 20.1 | 15022.45 |
| 27 | 24.7 | 15593.34 |
| 28 | 20.7 | 15983.31 |
| 29 | 24.3 | 15303.3 |
| 30 | 30.2 | 7694.417 |
| 31 | 30.53 | 5559.305 |
| 32 | 25.6 | 14097.22 |
| 33 | 26.6 | 11057.54 |
| 34 | 16.9 | 13914.78 |
| 35 | 26.4 | 14120.7 |
| 36 | 17 | 14127.47 |

**Table S4: Ct values of the clinical samples used in this study**

| Sars-CoV-2 | 1x (50uL reaction) | Final concentration |
| --- | --- | --- |
| RPA rehydration buffer | 29.5 | / |
| SS-IV | 0.5 | 2 units |
| Forward primer (25uM) CV125 | 1 | 500nM |
| Reverse primer (25uM) SC.B3 | 1 | 500nM |
| Cas12b (original stock) | 0.3 | 110nM |
| crRNA (5uM) CV456 | 0.5 | 100nM |
| Hex-reporter | 3.75 | 750nM |
| RNase H | 0.5 | 1 unit |
| H2O | 4.95 |  |
| Total | 42 |  |
| Divide equally into two tubes and add the following to initiate the reaction |  |  |
| RNA sample | 2 |  |
| Magnesium acetate (280mM) | 2 |  |

Volumes and final concentrations during optimization reactions varied as described in the results section.
